# Training for managing impacted fetal head at caesarean birth: multimethod evaluation of a pilot

**DOI:** 10.1136/bmjoq-2023-002340

**Published:** 2023-07-31

**Authors:** Jan W van der Scheer, Katie Cornthwaite, Pauline Hewitt, Rachna Bahl, Wendy Randall, Alison Powell, Akbar Ansari, Bothaina Attal, Janet Willars, Matthew Woodward, Imogen A F Brown, Annabelle Olsson, Natalie Richards, Evleen Price, Alessandra Giusti, Joann Leeding, Lisa Hinton, Jenni Burt, Mary Dixon-Woods, Giulia Maistrello, Nick Fahy, Oscar Lyons, ABC Contributor Group, Tim Draycott

**Affiliations:** 1 THIS Institute (The Healthcare Improvement Studies Institute), Department of Public Health and Primary Care, University of Cambridge, Cambridge, UK; 2 Royal College of Obstetricians and Gynaecologists, London, UK; 3 Translational Health Sciences, University of Bristol, Bristol, UK; 4 Royal College of Midwives, London, UK; 5 University Hospitals Bristol and Weston NHS Foundation Trust, Bristol, UK; 6 RAND Europe, Cambridge, UK; 7 Nuffield Department of Primary Care Health Sciences, University of Oxford, Oxford, UK; 8 North Bristol NHS Trust, Westbury on Trym, UK

**Keywords:** Cesarean delivery, Healthcare quality improvement, Medical education, Obstetrics and gynecology, Simulation

## Abstract

**Background:**

Implementation of national multiprofessional training for managing the obstetric emergency of impacted fetal head (IFH) at caesarean birth has potential to improve quality and safety in maternity care, but is currently lacking in the UK.

**Objectives:**

To evaluate a training package for managing IFH at caesarean birth with multiprofessional maternity teams.

**Methods:**

The training included an evidence-based lecture supported by an animated video showing management of IFH, followed by hands-on workshops and real-time simulations with use of a birth simulation trainer, augmented reality and management algorithms. Guided by the Kirkpatrick framework, we conducted a multimethod evaluation of the training with multiprofessional maternity teams. Participants rated post-training statements about relevance and helpfulness of the training and pre-training and post-training confidence in their knowledge and skills relating to IFH (7-point Likert scales, strongly disagree to strongly agree). An ethnographer recorded sociotechnical observations during the training. Participants provided feedback in post-training focus groups.

**Results:**

Participants (N=57) included 21 midwives, 25 obstetricians, 7 anaesthetists and 4 other professionals from five maternity units. Over 95% of participants agreed that the training was relevant and helpful for their clinical practice and improving outcomes following IFH. Confidence in technical and non-technical skills relating to managing IFH was variable before the training (5%–92% agreement with the pre-training statements), but improved in nearly all participants after the training (71%–100% agreement with the post-training statements). Participants and ethnographers reported that the training helped to: (i) better understand the complexity of IFH, (ii) recognise the need for multiprofessional training and management and (iii) optimise communication with those in labour and their birth partners.

**Conclusions:**

The evaluated training package can improve self-reported knowledge, skills and confidence of multiprofessional teams involved in management of IFH at caesarean birth. A larger-scale evaluation is required to validate these findings and establish how best to scale and implement the training.

**WHAT IS ALREADY KNOWN ON THIS TOPIC**

- Impacted fetal head (IFH) is a technically challenging obstetric emergency that may complicate up to 10% of caesarean births in the UK and is associated with potentially devastating complications for mother and baby.
- Recent surveys of UK maternity professionals indicate a lack of confidence and underuse of appropriate techniques for managing IFH, in part because of paucity of high-quality training.

**WHAT THIS STUDY ADDS**

- Multiprofessional training including lecture-based, visual and simulation methods can improve self-reported technical and non-technical knowledge and skills of maternity professionals for anticipating, identifying and managing an IFH.

**HOW THIS STUDY MIGHT AFFECT RESEARCH, PRACTICE OR POLICY**

- The training package is ready for use across the UK following validation in a larger, representative sample of UK maternity units, including establishing how best to scale and implement the training at a local and regional level.
- Systematic monitoring will be needed to determine long-term effectiveness of implementation of the training.

## Introduction

Impacted fetal head (IFH) is a technically challenging obstetric emergency increasingly encountered during unplanned caesarean births.^1–3^ Complicating up to 10% of caesarean births in the UK (1.5% of all births),^4–6^ it is associated with significant risks to mother and baby, including postpartum haemorrhage, trauma to uterus and bladder, hypoxic ischaemic brain injury and skull fractures.^1 2 4 6^ Reports of perinatal brain injury linked to IFH have risen in recent years, along with coronial inquiries and increased litigation nationally and internationally.^6–10^ In recent surveys, UK maternity professionals reported lack of confidence, knowledge and skills relating to anticipating, identifying and managing an IFH, possibly owing to paucity of and limited access to high-quality training.^11–14^ As a result, practice in relation to IFH management is variable,^12 15^ likely contributing to adverse outcomes.^6^

Although the need for IFH training was identified over 10 years ago,^16^ the UK currently lacks nationally standardised, multiprofessional training aimed at improving the technical skills (table 1)^1 17–22^ and non-technical skills (eg, teamwork, communication)^23–26^ required for managing IFH. In management of other obstetric emergencies, implementation of high-quality training that includes simulation-based practice has been associated with significant improvement in clinical outcomes.^27^ Simulation-based practice is likely to be particularly important for IFH training,^28 29^ since efforts to ensure workplace learning during clinical care are challenged by various factors. These factors include the unpredictability of IFH, variable availability of experienced obstetricians to supervise trainees and difficulties in demonstrating the techniques executed below the level of incision where they cannot be seen.^4 12 15^

**Table 1:** The range of techniques that may be employed to prevent and manage impacted fetal head (IFH) at caesarean birth^1 17–22^

Techniques for prevention of IFH (prior to starting caesarean birth)	
Manual vaginal disimpaction (‘push-up’)	Introducing a hand into the vagina to move the head up into the abdomen prior to making a uterine incision to reduce likelihood of IFH.
Fetal pillow(R)	Using an inflatable device in the vagina to move the head up into the abdomen prior to making a uterine incision to reduce likelihood of IFH.

Techniques for management (when encountered IFH during caesarean birth)	
Manual abdominal cephalic disimpaction	Using the dominant or non-dominant hand to flex and lift the baby’s head upwards into the maternal abdomen to deliver the head.
Manual vaginal disimpaction (‘push-up’)	Introducing a hand into the vagina to move the head up into the abdomen when encountered IFH at caesarean birth.
Reverse breech extraction	A hand is introduced in the upper aspect of the uterus, the baby’s feet are grasped and the baby is delivered feet first (breech). Once the baby’s shoulders are delivered, the head is lifted out of the pelvis.
Patwardhan method	A modification of reverse breech extraction, whereby the arms are delivered first.
Tocolysis	Administration of medicine (tocolysis) to relax the uterus and facilitate advanced disimpaction techniques.

Despite the need for practice of techniques to prevent or manage IFH (table 1),^22^ training tested so far has not included birth simulators that enable realistic rehearsal of techniques such as vaginal disimpaction and reverse breech extraction.^30–33^ Current training is also limited by a tendency to focus exclusively on obstetricians.^30–33^ This neglects the key role of midwives in performing vaginal disimpaction^15^ and misses opportunities to foster multiprofessional teamwork, communication with those in labour and their birth partners, and other non-technical skills for managing obstetric emergencies.^23–26^

In this article, we report an evaluation of a multiprofessional training package for managing IFH at caesarean birth as part of the Avoiding Brain Injury in Childbirth (ABC) programme, which was commissioned by the UK’s Department of Health and Social Care in 2021.

## Methods

### Training package

We developed a training package, informed by national surveys on IFH practice and training,^12 14^ development and validation of a novel birth simulator,^15^ and systematic review and appraisal of literature on IFH management.^22 34^ Development included codesign,^35–37^ with maternity professionals and service users, of an evidence-based lecture, hands-on workshops and simulated scenarios. These were supported by IFH management algorithms developed based on user testing and qualitative research with maternity professionals.^22 34^ Patient and public involvement (PPI) informed principles for communication with women and birth partners during obstetric emergencies, which were applied throughout the training, including in the algorithms. Online supplemental material 1 provides a summary of key learning points for participants and the communication principles. Table 2 provides an overview of the training.

10.1136/bmjoq-2023-002340.supp2 Supplementary data

**Table 2:** Overview of the training for anticipating, identifying and managing impacted fetal head (IFH)

Lecture and video (55 min)	
Training presentation (45 min)	The training presentation described the problem of IFH at caesarean birth and outlined the evidence base on how to manage it. The presentation contained information about the definition and incidence of IFH, complications and risk factors for IFH at caesarean birth and good practice in communication with people in labour and birth partners. The presentation also introduced the four IFH management algorithms used during workshops and training (ie, a master algorithm detailing overall management and three separate algorithms providing details of how to perform abdominal cephalic disimpaction, vaginal disimpaction and reverse breech extraction).
Animated video (10 min)	The IFH animated video (see figure 1) demonstrates the clinical manoeuvres for abdominal cephalic disimpaction, vaginal disimpaction and reverse breech extraction.

Workshops (60 min)	
Workshop 1: abdominal cephalic disimpaction (20 min)	This workshop was designed for obstetricians to practise abdominal cephalic disimpaction and for other participants to observe and gain more understanding of the problem. The trainer demonstrated abdominal cephalic disimpaction using the PROMPT Flex simulator and referring to the relevant IFH algorithm. Obstetricians were able to practise abdominal cephalic disimpaction of at least one fetal head position (occipito posterior, occipito transverse and/or occipito anterior).
Workshop 2: vaginal disimpaction (20 min)	This workshop was designed for both obstetricians and midwives to practise vaginal disimpaction methods and for other participants to learn about the positioning of the person in labour to undertake these manoeuvres. The trainer showed participants how to reposition the legs of the woman. Participants learnt and practised how to gain vaginal access safely and effectively and how to cradle, flex and elevate the baby’s head.
Workshop 3: reverse breech extraction (20 min)	This workshop was designed for obstetricians to practise reverse breech extraction and for other participants to observe and gain more understanding of the problem. A trainer demonstrated reverse breech extraction using the PROMPT Flex simulator and referring to the relevant IFH algorithm. Obstetricians were able to practise reverse breech extraction of at least one fetal head position (occipito posterior, occipito transverse and/or occipito anterior). The demonstration included a brief overview of how to perform an inverted T or J shaped uterine incision.

Simulation (40 min)	
Simulation pre- briefing (5 min)	The trainer explained to all participants the setting for the simulation and the location of equipment, management algorithms, emergency telephone, glyceryl trinitrate spray, etc. The trainer briefed the ‘pregnant’ actor before the simulation (see online supplemental material 1). The trainer reminded participants to refer to the algorithms for IFH at caesarean birth and emphasised that participating in the simulation is not a test. Participants were given time to familiarise themselves with the simulation setting and to ask any questions.
Simulation briefing, role allocation and checklists (10 min)	The trainer introduced the simulation, ensuring all participants were aware of their roles and were familiar with the equipment and answering any participant questions before the simulation started. The trainer identified the multiprofessional simulation participants. The simulation started with the following roles allocated: an obstetrician, operating assistant, anaesthetist, midwife and circulating theatre nurse/maternity support worker. The trainer distributes checklists to observing team members, that is, a clinical checklist, teamwork checklists (communication, team roles and leadership, situational awareness) and a ‘pregnant’ actor checklist. The trainer provided simulation information (online supplemental material 1) to the theatre team using situation, background, assessment and recommendation (SBAR).
Real-time simulation with multiprofessional team (10 min)	This simulation enabled the participating team to practise managing IFH at caesarean birth in real time. The simulation focused on the non-technical skills required to manage IFH, including communication, teamwork and situational awareness, as well as the technical and practical aspects of safe and effective clinical management. The trainer paused or stopped the simulation at any point if the team was going significantly off track. For example, if the team made repeated attempts at one approach to disimpaction and was not able to deliver the baby, the trainer asked the team to pause and reconsider their approach and then restart the simulation. If non-recommended and potentially unsafe techniques, such as a single forceps blade, were requested or employed, the trainer stopped the simulation, explained why and then restarted the simulation.
Simulation debrief (10 min)	The trainer asked the team members how they felt the simulation went and asked the observers to give feedback using their checklists, using the following questions—asked to clinical simulation participants, observers and/or ‘pregnant’ actors and birth partner—to guide the debrief: How do you feel that went? What was done particularly effectively? What do you think should have been done differently? Would you like me to explain what you might have done differently?
Finish (5 min)	The trainer emphasised the importance of good teamworking and communication and how this can influence the team culture, reminding participants that the most effective clinical teams declare the emergency and use closed-loop communication. The trainer summarised the key clinical learning points from the simulation and referred to the use of the clinical algorithms. The trainer ensured the discussion also included how the team explained the emergency to the person in labour and their birth partner and the importance of debriefing afterwards.

**Figure 1:**
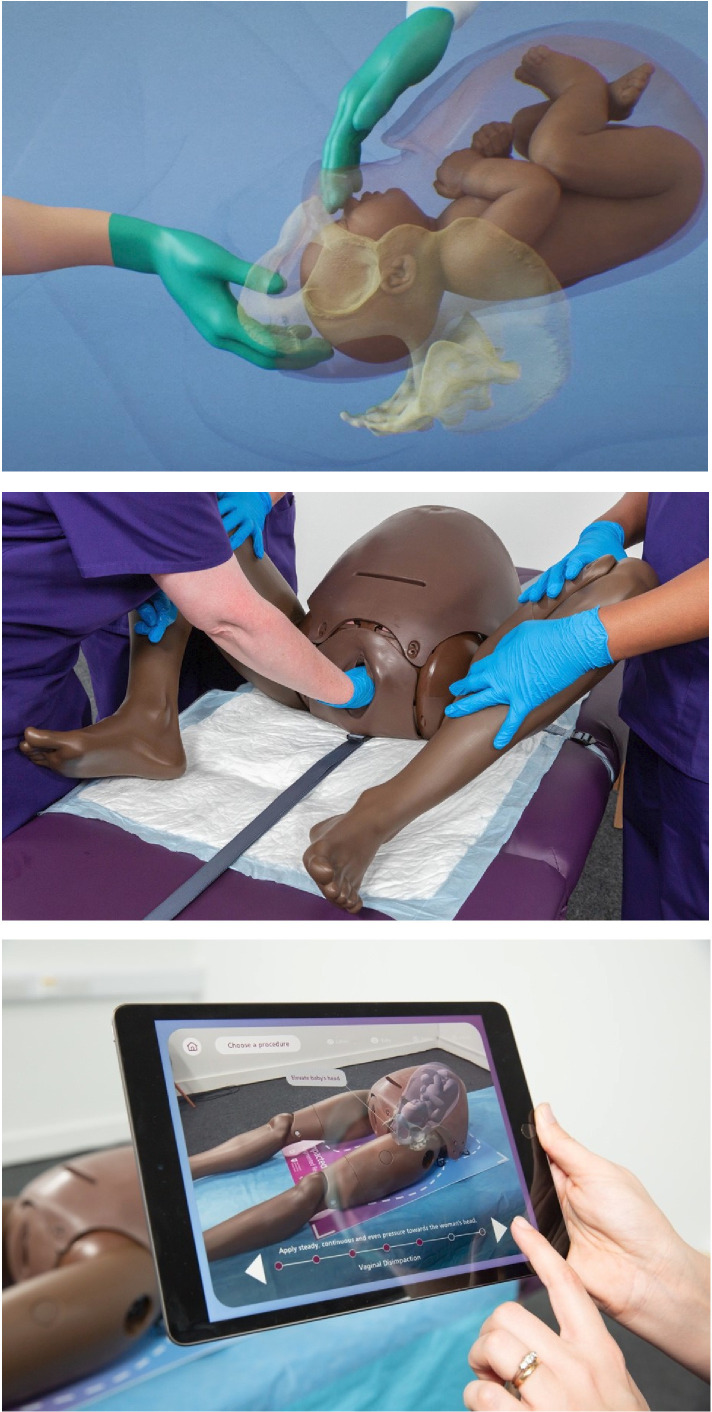
Animation video (screenshot, top image), birth simulation trainer with practice of vaginal disimpaction (photo, middle image) and augmented reality tool (photo, bottom image) as used during the training.

Training was designed for delivery in a single session lasting up to 3 hours. The session started with a lecture presenting evidence on anticipating, identifying and managing an IFH (table 2). The lecture introduced the communication principles (online supplemental material 1), including examples of ‘conversation starters’ for healthcare professionals communicating during obstetric emergencies (eg, “Impacted fetal head means that the baby’s head is wedged low in your pelvis. We have a plan to manage this and some colleagues will arrive to assist with the birth.”) An animated video visualised anatomical considerations for IFH and illustrated how to perform manoeuvres to deliver an IFH at caesarean birth (figure 1 and table 2). In the pilot testing (see below), the lecture and video were only available to participants during the session.

Next, the participating teams took part in three subsequent workshops (table 2). During the workshops, participants obtained hands-on training of all disimpaction techniques using the management algorithms, augmented reality tool and validated birth simulation trainer (figure 1).^15 34^

Finally, the teams participated in a real-time standardised, simulated scenario of IFH during caesarean birth (online supplemental material 1). This enabled further practice of technical skills for IFH management techniques (tables 1 and 2) and non-technical skills such as multiprofessional teamwork, team communication and application of the principles for communicating with women and birth partners (table 2 and online supplemental material 1). The simulations included a ‘pregnant’ actor and birth partner (see PPI section below), who received a standardised briefing before the simulation (online supplemental material 1). The training finished with a clinical debrief, consistent with recommended simulation practice.^28^

### Patient and public involvement (PPI)

PPI was focused on communication with women and birth partners during obstetric emergencies. Five service users who had a range of maternity experiences and experience of advocating for improvement and inclusion of under-represented voices, provided PPI input to the overall ABC programme. This group helped shape the training package during fortnightly meetings. Additional input was gained from a group of 15 women with experience of obstetric emergency, who met twice online in facilitated focus groups to discuss communication during obstetric emergencies, with focus on IFH. We also invited representatives of Maternity Voices Partnerships (MVP)^38^ to attend the training as an observer or simulation actor and to provide feedback in the post-training focus groups (see below). All MVP representatives provided written consent to take part in the quality improvement activity.^39^ The PPI contributors were invited to join the ABC Contributor Group for academic outputs and were paid in accordance with National Institute for Health and Care Research guidance on involvement.^40^

### Multimethod evaluation

The training was piloted with multiprofessional teams in sessions facilitated by ABC clinicians (KC, PH, RB, WR). We conducted a multimethod evaluation of the training, guided by the Kirkpatrick framework.^41–43^ The evaluation focused on the first two Kirkpatrick levels: (1) participants’ reactions to and opinions about the training and (2) the extent to which participants acquired new knowledge, skills, attitudes, confidence and commitment based on their participation in the training.^42^ Similar methodology has been used to evaluate training for other obstetric emergencies.^44–46^ The evaluation was a quality improvement activity,^39 47^ including observations, focus group discussions and questionnaires.

#### Settings and participants

The training involved the range of healthcare professionals who are likely to be present in the operating theatre at the time of caesarean births in UK maternity units (eg, midwives, obstetricians, anaesthetists, maternity support workers, theatre staff). Due to ongoing pressures caused by the COVID-19 pandemic, the five participating units were selected primarily on their availability and ability to facilitate simulation sessions. They did represent diversity of maternity settings. Local training leads in each unit invited relevant team members. Interested and available team members provided written consent to take part and agreed to recording of the quality improvement activity.^39^ Participants were offered a £50 thank you voucher and invited to join the ABC Contributor Group for academic outputs.

#### Observations

A trained ethnographer with experience in studying maternity care observed the training sessions, using a fieldnote form to characterise relevant aspects of the observed sociotechnical system, focusing on non-technical skills (online supplemental material 2). This included observations on teamwork during IFH management, professional roles and boundaries, communication among team members, communication of team members with the ‘pregnant’ actor and birth partner, as well as the atmosphere during the sessions. The ethnographer augmented their handwritten fieldnotes following the session and dictated them for audio transcription.

10.1136/bmjoq-2023-002340.supp3 Supplementary data

#### Focus groups

Immediately after the training, the ethnographer facilitated an audio-recorded focus group discussion (approximately 30 min) based on a semistructured topic guide (online supplemental material 2). Discussion focused on: (i) what participants perceived as the most and least helpful parts of the training, (ii) how teamwork might change as a result of the training and (iii) potential impact of the training on communication with women and birth partners during IFH emergencies. Service user representatives who observed or acted during the training participated in the discussions (see PPI section above). Audio recordings were transcribed verbatim.

#### Questionnaires

**Figure 2:**
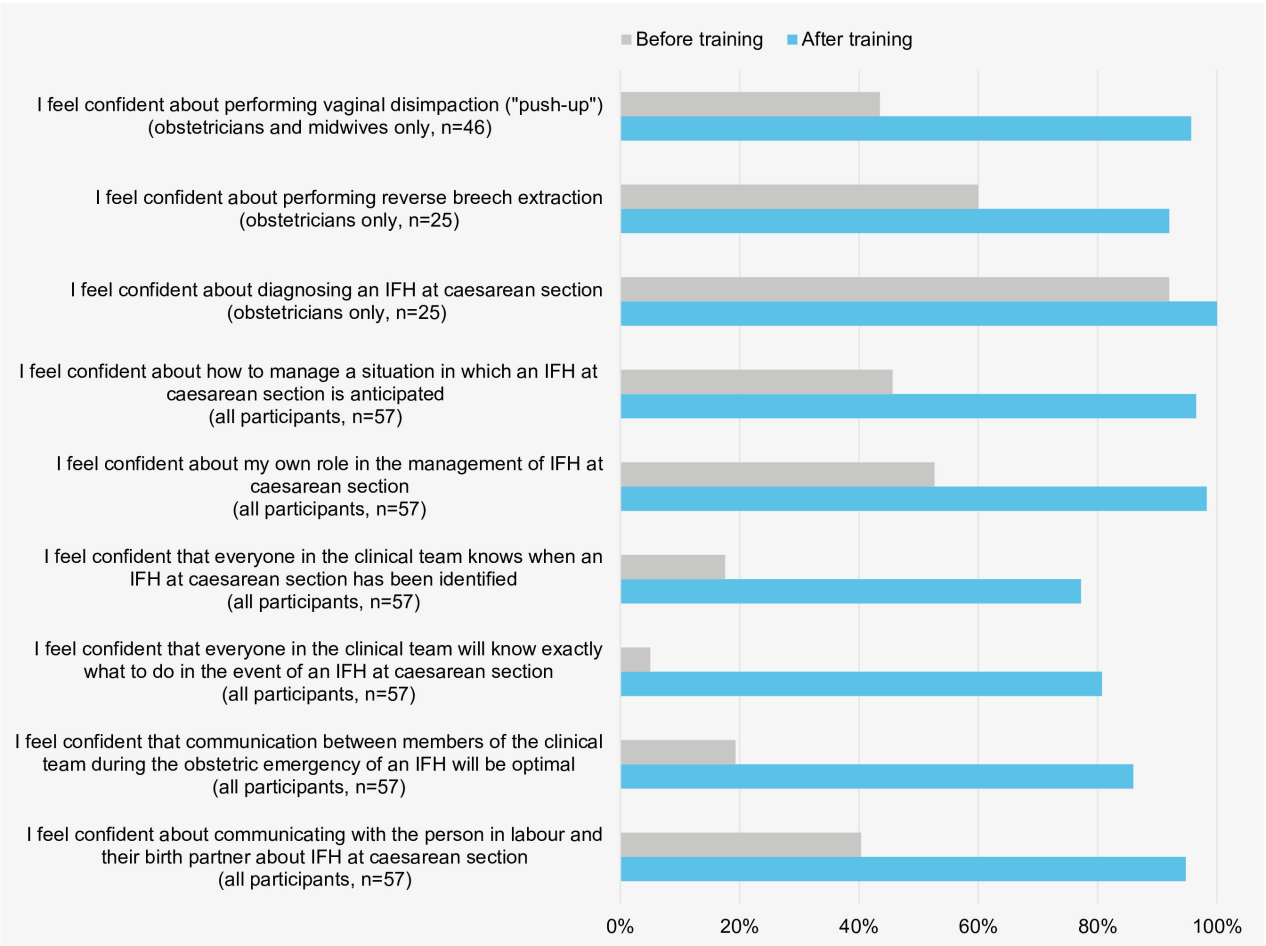
Percentage of participants, before and after training, who strongly agreed or agreed with statements on confidence in knowledge and technical/non-technical skills relating to anticipating, identifying and managing impacted fetal head (IFH) at caesarean birth. Online supplemental table S2 provides a detailed breakdown of the data.

Online questionnaires (online supplemental material 2) were administered immediately before the training and immediately after the focus group discussions. Participants completed the questionnaires using a Qualtrics weblink accessed through their mobile devices. Before and after training, participants rated statements about confidence in knowledge and technical/non-technical skills addressed in the training (figure 2 and online supplemental table S1). Ratings were given along 7-point Likert scales, ranging from strongly agree to strongly disagree. The post-training questionnaire additionally asked participants to rate statements (along 7-point Likert scales) on how helpful or useful the training was to their clinical practice and improving outcomes following IFH (table 3 and online supplemental table S2). It also asked about the extent to which they would recommend the training to a colleague (Net Promoter Score, scale of 1–10).^48^

10.1136/bmjoq-2023-002340.supp1 Supplementary data

**Table 3:** Number and percentage of participants agreeing with post-training questionnaire statements on relevance and helpfulness of the training for anticipating, identifying and managing impacted fetal head (IFH)

Post-training questionnaire statement	Strongly agree or agree
This training was relevant to my clinical practice in relation to managing IFH at caesarean section	56 (98%)
The training will help to improve outcomes following IFH at caesarean section	55 (96%)
The training will help maternity staff better manage IFH at caesarean section	55 (96%)
The simulations helped my learning in the management of IFH at caesarean section	57 (100%)
The use of augmented reality helped my learning in managing IFH at caesarean section	40 (70%)
The animated video helped my learning in managing IFH at caesarean section	55 (96%)
The management algorithms will support my clinical practice or participation in managing IFH at caesarean section	55 (96%)
This training overall will improve my clinical practice or participation in managing IFH at caesarean section	56 (98%)

Online supplemental table S1 provides a detailed breakdown of the data.

### Data analysis

We analysed the questionnaire data using descriptive statistics, only including data of participants who completed the pretraining and post-training questionnaires (complete-case analysis excluding incomplete data of five participants). For the Net Promoter Score, we calculated the ratio of promoters (score of 9 or 10) to detractors (score of 0–6).^48^ For the questions on confidence in knowledge and skills, we determined the mean of difference in scores before and after training (post−pre mean). The small sample size precluded any statistical comparisons.

We analysed the transcribed observation notes and focus group discussions to generate initial common themes with a focus on: (i) participants’ reactions to and opinions about the training and (ii) the extent to which participants perceived that they had acquired new knowledge or skills.^41–43^ Building on these initial themes, we then undertook further analysis^49^ to generate further insight into how the training had been received and potential areas for improvement. Themes identified overlapped with three main questionnaire topics (ie, understanding of IFH, multiprofessional teamwork, communication). These three topics guided the final synthesis of the observation and focus group data, which was refined in discussion with the wider multidisciplinary evaluation team.

## Results

### Settings and participants

Pilot testing and evaluation of the training took place with 57 multiprofessional team members from five different units (21 midwives, 25 obstetricians, 7 anaesthetists and 4 other professionals). The five units included tertiary and district general hospitals from different UK regions (East England, South East England, West England), ranging from 3000 to 8000 births per year, all with obstetric-led and alongside midwifery-led settings. Participants from two units took part in pilot testing in their own clinical settings, while the other three units were facilitated at a specialist venue with a clinical simulation space. Four MVP representatives observed and/or acted during one or more training sessions.

### Questionnaires

Responses to the questionnaires suggested very positive participant reactions to the training. Over 95% of participants agreed that the training was relevant and helpful for their clinical practice and improving outcomes following IFH (table 3 and online supplemental table S2). We found very high levels of participant agreement that learning was aided by the simulations (100%, n=57), management algorithms (96%, n=55) and animated video (96%, n=55) (table 3 and online supplemental table S2). The Net Promoter Score was very high (88%), with 50 participants giving a rating of 9–10 and others giving a rating of 7–8 (n=6) or 5 (n=1).

The questionnaires also suggested that participants acquired new knowledge and technical/non-technical skills. Self-reported confidence in knowledge and skills relating to managing IFH at caesarean birth was variable before the training, but improved in nearly all participants after the training (figure 2 and online supplemental table S2). The largest increases in confidence (post−pre mean) were observed in the technical skill of performing vaginal disimpaction (+1.76), the non-technical skills of teamwork (+2.51), communication among team members (+1.77) and communicating with those in labour and their birth partners (+1.61) (online supplemental table S2).

### Observations and focus groups

The observation and focus group data allowed insight into the role of the training in better understanding how to manage IFH in clinical practice.

#### Better understanding

The focus groups highlighted that participants felt the training addressed an important gap for some. For others, the training formalised and extended knowledge and technical skills that had been gathered experientially.

I’ve been a midwife for over 20 years and I cannot believe I have not been taught how to disimpact a fetal head when it is so important. (Midwife)

It’s just things that you have learned over time, over experiences and everything. But you now have the format and a clear picture in your head. (Obstetrician)

Midwifery participants explained that the training helped to better understand the severity and complexity of IFH and how the theory and practice introduced in the training increased confidence in technical skills such as performing vaginal disimpaction. Obstetricians also noted how the training had enhanced their technical knowledge and skills.

The situation and the practice that we did was really good, it kind of complemented what I already knew and kind of polished me a bit. (Obstetrician)

The animated video, birth simulator and augmented reality tool helped uncover details of an emergency that in daily practice remains largely ‘hidden’. Participants discussed the importance of both obstetric and non-obstetric team members (eg, midwives, anaesthetists, other theatre staff) seeing the specifics of IFH and its management in a manner not possible in practice.

They [an obstetrician] also made the point [during a workshop] that it was really important to have appropriate training and to be competent in this procedure and that it was quite a difficult thing to learn because it was so hidden. Therefore, they said that the video […] had been very helpful in helping to show what was going on inside. (Ethnography notes)

[…] it’s a lot about the visualisation of what’s actually happening with impacted fetal head because a lot of the MDT team, […] theatre team and anaesthetists don’t really know what’s going on with that hand and that head and that. Showing that visually really helped. (Obstetrician)

#### Multiprofessional teamwork

The training appeared to improve non-technical skills around teamwork, in part by enhancing the understanding of the different roles and contributions of team members. This included further appreciation of all team members of what obstetricians need to do during an IFH emergency and their challenge of finding the right technique for the specifics of the presenting clinical situation.

[Practising together] gave a wider appreciation to the difficulties faced by the obstetricians, that it is not just an obstetrician problem and that everybody else in the room can help. (Ethnography notes)

You’ve just got a better understanding of what the obstetrician’s going through, it’s not, oh come on, deliver this baby. You're actually like, they’re trying. But they’re trying to find the right technique in…to delivery. (Midwife)

Similarly, an ethnographer noted “[it] was a bit of a revelation to a few” that when the obstetrician paused to allow the uterus to relax after a contraction induced by the obstetrician’s hand being placed inside the uterus, this was a deliberate pause and not a moment of indecision. Such ‘revelations’ were facilitated by working in smaller groups and sequential workshops to allow various team members to experience techniques hands-on.

The anaesthetist was overheard saying […] he had been oblivious to what that [vaginal disimpaction] meant, he thought that it was just a push and that there was no skill to it. Felt he had had his eyes opened to the complexity of the procedures that were involved in disimpacting a fetal head. (Ethnography notes)

[…] there is real value for midwifery there to understand […] getting hands on in the sim, to actually understand what that obstetrician is going through. Trying to get that hand under that fetal head was really powerful for me. (Midwife)

Training led to better understanding of each other’s roles and improved awareness that managing IFH crosses traditional professional boundaries and requires inclusion of all multiprofessional members to maximise learning across the theatre team.

[considering] past history of maternity and theatre teams having issues […], a lot of the guys think that as soon as you hit those doors into theatre that’s their area. (Theatre practitioner) And it’s a shame, because if we were trained together then we would break down some of those barriers, wouldn’t we? (Midwife) Exactly. (Theatre practitioner)

They [maternity support workers] are actually really fundamental to this because they will literally be picking stuff up, putting the calls out, you know. (Midwife)

The management algorithms were seen as helpful in enabling teams to create a shared mental model of management steps and in facilitating consultant obstetricians in rapidly gaining an overview of the obstetric emergency if they enter the theatre at a later stage.

That is the benefit of the multidisciplinary training, knowing that you’re working your way through an algorithm […] If this doesn’t work, we do this […] that we all understand and that we’re all trained to do the same. (Obstetrician)

[…] so often we as consultants get called not from the beginning. So, what’s really helpful to know is what’s already been tried […] you know where you are in the algorithm and you know what they’ve tried. (Obstetrician)

#### Communication

Training in non-technical skills around team communication helped participants realise how important verbalisations in theatre were, such as stating the clinical situation is an IFH emergency and what clinical action is being undertaken. Participants also positively reflected on the training’s emphasis on high quality communication with those in labour and birth partners in all aspects of the training.

…whenever we do skills training or [name of training], it’s always mentioned but it’s almost, sort of, mentioned as a throwaway comment, you know, oh and of course you were…debrief your woman or…it’s almost, like, tagged on at the end. So it’s the first time I’ve really experienced where it [communication] had a real showcase. (Midwife)

Participants found the conversation starters from the lecture a particularly useful resource in supporting better communication with the women and birth partners during the emergency.

It’s given me quite a lot to take away to the other emergencies that we manage, PPHs [post-partum haemorrhages] where the caesarean takes a long time or shoulders [dystocia] is a good example where you might need to, sort of, communicate. Yeah, I agree, the conversation starter sentences are really good. (Obstetrician)

MVP representatives highlighted during the focus groups the importance of communication with the woman about the possibility of vaginal disimpaction, obtaining informed consent for vaginal disimpaction, and the potential negative effects of silences during critical moments (eg, feeling stressed about whether a silence indicates clinicians’ anxiety and/or a negative outcome). They recommended involving service users in the further design and evaluation of the training. During the focus groups, participants and MVP representatives reflected on the importance of finding the right balance of communicating with the person in labour and their partner, while remaining focused on the technical manoeuvres required to safely deliver the baby. Participants and MVP representatives generally agreed that the training could further emphasise that one professional (eg, the anaesthetist) should be designated to communicate with the woman and birth partner during the emergency. Two obstetricians felt that the priority should be given to clinical manoeuvres while expressing that an explanation would follow as soon as the acute emergency was more under control.

## Discussion

In this evaluation of a training package for managing IFH at caesarean birth involving 57 multiprofessional team members from five UK maternity units, the vast majority of participants considered the training relevant and helpful for their clinical practice and improving outcomes following IFH. Participants reported substantial improvements in confidence in knowledge and skills for the technical and non-technical aspects of anticipating, identifying and managing an IFH. Supported by a range of resources and learning methods, the training appeared to enhance three key areas of understanding and practice. First, participants better understood the complexity of IFH by observing and experiencing the otherwise ‘hidden’ aspects of IFH and its management techniques. Second, training as a multiprofessional team improved non-technical skills, including better awareness that managing IFH requires a co-ordinated team effort including the obstetrician, midwife, anaesthetist and other team members. Third, participants furthered their understanding of optimal communication during the emergency, including the benefits of standardised communication among team members and the value of bespoke communication with women and birth partners.

Our findings of improved confidence in technical skills indicate that the training familiarises maternity professionals with IFH techniques in simulation. This could help address inexperience and feelings of uncertainty reported by UK maternity professionals when needing to use a variety of techniques to prevent or manage IFH.^12 14^ Indeed, midwifery participants in particular expressed surprise and dissatisfaction about lack of previous training in this area, as also found in previous surveys.^12 13^ The training substantially increased participants’ confidence in the technique of vaginal disimpaction (‘push-up’), potentially reducing the risk of neonatal injury attributed to incorrect execution of this technique.^9 50^ The training is also the first to provide realistic hands-on practice and improve confidence of obstetricians in reverse breech extraction, a technique likely underused in UK practice due to inexperience.^12^ This was enabled by the use of a novel validated birth simulation trainer, which provides more opportunities for practising caesarean birth techniques than other available birth trainers.^12 15 34^ Although further evaluation is required, improved confidence of maternity professionals in their technical IFH skills could help reduce current variation in practice^12 15^ and risks to mother and baby associated with IFH.^1 2 4 6^

The improved confidence in team management for IFH found in this evaluation is consistent with previous findings of the benefits of multiprofessional simulation training for obstetric emergencies.^27 51–54^ IFH technical training that focuses on obstetricians alone^30–33^ is unlikely to deliver the improvement needed in required non-technical skills associated with multiprofessional training. Required non-technical skills include those relating to anticipation and preparation, situation awareness, communication and shared understanding, teamwork and behaviour, as well as decision-making.^23–26 55^ Obstetrician-only training also overlooks the potentially beneficial effects of multiprofessional simulation practice on a collaborative culture and psychological safety.^28 56 57^

Participants found the IFH management algorithms helpful for creating shared mental models for the team when dealing with the emergency. Again, this finding aligns with practice in other areas of obstetrics.^58–60^ Examples include shoulder dystocia and assisted vaginal birth, where structured task lists or algorithms have also successfully been used to transfer knowledge across a maternity team, both in real life and simulation.^58–60^ The management algorithms were grounded in evidence from a systematic review, PPI feedback and clinical user testing,^22 34^ in contrast to previously proposed algorithms.^32 61 62^

The improved confidence in team communication for IFH was likely related to the training’s emphasis on standardised use of language. This finding is similar to evidence on other obstetric emergencies that for example shows that clearly and calmly declaring the emergency using unambiguous terminology facilitates teamworking, communication and management.^23 26 63^ An important element of the training was its emphasis on communicating with service users during emergency healthcare provision.^64–68^ Participants valued this element of the training, such as the opportunity to practise using the communication principles and conversation starters that had been codesigned with the PPI group. Any future development or piloting of the training should continue with input from service users,^69–71^ as emphasised by MVP representatives during the focus group discussions.

### Strengths and limitations

This is the first evaluation of multiprofessional training for managing IFH at caesarean birth using lecture-based, visual and simulation methods to maximise learning of maternity professionals and teams. It is distinct from previous work on IFH training that focused on obstetricians alone or was limited by the surgical techniques taught.^30–33^ A further strength of the evaluation is the inclusion of various types and sizes of hospitals, maternity professionals with different levels of experience and input from maternity service users. Other strengths include use of a structured framework (Kirkpatrick) to inform the multimethod evaluation and the multidisciplinary data collection and analysis.

This evaluation does have some limitations. Ongoing pressures caused by the COVID-19 pandemic required the use of convenience sampling of units and piloting of the training with a relatively small participant sample. This might limit representativeness of our results for the wider population of maternity units and professionals in the UK. Another limitation is the use of complete-case analysis for the questionnaires, which may have introduced some biased estimates if there were differential responses to particular questionnaire items.^72^ Although it was not possible to quantify whether any social desirability bias may have affected scores, the observations and focus groups showed no indication of this. Finally, the scope of this evaluation did not stretch to evaluating evidence for changes in clinical outcomes, though it is promising that over 95% of participants agreed that the training would help improve outcomes following IFH.

## Conclusion

Multiprofessional training for IFH at caesarean birth, using high-quality training methods including simulation, has very promising potential to improve maternity professionals’ knowledge and skills for managing IFH. Validation of our findings is needed in a larger, representative sample of UK maternity units, including establishing how best to scale and implement the training at a local and regional level. This should be followed by national dissemination and implementation of the training and evaluation of its impact using a core outcome set or routine data collection of IFH-specific clinical outcomes.

## Data Availability

Data are available upon reasonable request. The data that support the findings of this evaluation are available on request from the corresponding author. Access to fully anonymised data may be granted to bona fide researchers under a data sharing agreement. The data are not publicly available due to privacy or ethical restrictions.
