# Supplementary material for: Training for managing impacted fetal head at caesarean birth: multimethod evaluation of a pilot
Source: BMJ Open Qual. 2023 Jul 31;12(3):e002340. doi: 10.1136/bmjoq-2023-002340 (PMC10391817; doi:10.1136/bmjoq-2023-002340)
Supplement: Supplementary data [file bmjoq-2023-002340supp002.pdf]

Supplementary Material 1

Contents

Supplementary Material 1 1

Summary of key learning points of the training 2

ABC principles for communicating with women and birth partners 3

Simulation script described with SBAR (Situation, Background, Assessment, Recommendation) terminology 4

'Pregnant' actor instructions for the simulation 5

Summary of key learning points of the training

The aim is that those completing the training on impacted fetal head at caesarean birth using the ABC approach will:

- Recognise that impacted fetal head at caesarean birth is diagnosed when routine techniques used to deliver the fetal head at caesarean birth have been unsuccessful.
- Recognise that impacted fetal head at caesarean birth is as common before full cervical dilatation as it is at full dilatation. Obstetricians should therefore be prepared to encounter impacted fetal head at any emergency caesarean birth.
- Anticipate the possibility of impacted fetal head at caesarean birth if labour has been augmented with oxytocin and/or if features of obstructed labour are present, for example caput or moulding.
- Be particularly prepared for impacted fetal head when caesarean birth is performed at full cervical dilatation, when caesarean birth follows a failed assisted vaginal birth and/or where the fetal station is low.
- Be aware that the theatre team should be alerted if impacted fetal head at caesarean birth is anticipated.
- Consider disimpacting the head vaginally (manually or using a Fetal Pillow®) before starting the caesarean birth if the maternity team anticipates an impacted fetal head.
- Clearly and calmly declare ‘impacted fetal head’ and request help if the obstetrician is unable to disimpact the fetal head using standard delivery manoeuvres.
- Recognise the need to pause to allow relaxation of the uterus and to consider next steps.
- For abdominal cephalic disimpaction, be able to demonstrate understanding that:
    - if the obstetrician is unable to insert their hand anteriorly between the fetal head and pubic symphysis, there may be more space to get below the fetal head laterally
    - the operating obstetrician should keep their wrist straight and arm in the midline. The obstetrician may need to alter their position to achieve this
    - flexion of the fetal head is key – the direction of elevation should be towards the head of the woman, until the head is disimpacted.
- For vaginal disimpaction, be able to demonstrate understanding that:
    - the legs of the woman should be flexed and abducted to achieve adequate access, with two members of the maternity team supporting the legs at all times
    - flexion of the fetal head is key
    - the assistant performing vaginal disimpaction should use their whole hand to cup, flex and elevate the fetal head.
- For reverse breech extraction, demonstrate understanding that any pressure on the baby’s abdomen should be avoided.
- Be prepared to change their approach if no progress is achieved with a particular strategy.
- Document whether impacted fetal head was diagnosed and, if so, record all disimpaction techniques used.
- Use the ABC communication principles (**see below**) to ensure that women and their birth partners are included as members of the team, are listened to and are involved in discussions.

van der Scheer JW, *et al. BMJ Open Qual* 2024; 12:e002340. doi: 10.1136/bmjoq-2023-002340

ABC principles for communicating with women and birth partners

In consultation with women and birth partners, the ABC collaboration has co-designed three good practice principles for communication with women and birth partners in the obstetric emergency of impacted fetal head. These relate to: communication, language and including birth partners. The principles are attentive to diversity and inclusion.

Communication

- Allocate a team member to take the lead for communicating with the woman and their birth partner.
- Explain that impacted fetal head is a potential complication at caesarean birth.
- Explain the possible need for vaginal manoeuvres.
- Make sure you understand what is happening and can explain it to the woman and their birth partner.
- Be mindful that conversations in earshot of the woman and their birth partner can be distressing or anxiety-provoking.
- Non-verbal communication is important.
- Silence can cause the woman and their birth partner to think the worst.

Language

- Keep it simple.
- Women and their birth partners know that you may need to use medical terminology, but be aware that it can be excluding or upsetting.
- Be prepared to ‘translate’ medical terminology.

Including birth partners

- The person in labour and their birth partner are members of the team.
- Birth partners can help with communication.
- Include birth partners in discussions.
- Acknowledge that caesarean birth can be traumatic for the birth partner as well.

van der Scheer JW, *et al. BMJ Open Qual* 2024; 12:e002340. doi: 10.1136/bmjoq-2023-002340

Simulation script described with SBAR (Situation, Background, Assessment, Recommendation) terminology

**S**ituation	As a team, you are in theatre for an emergency caesarean birth because cervical dilatation has remained at 9cm. The situation has been explained to Meena and her birth partner, and Meena has given consent for caesarean birth.
**B**ackground	Meena is 40 weeks gestation in her first pregnancy, which has been uncomplicated. She started labour in the birth suite but was transferred to the delivery suite for oxytocin augmentation. Cervical dilatation has not changed for the past four hours, so after discussion with Meena, the obstetrician decides to perform a category 2 emergency caesarean birth. The cardiotocograph (CTG) is normal.
**A**ssessment	Meena has a good working epidural in situ. She has just had a vaginal examination in theatre, which showed no change in cervical dilatation and confirmed caput, moulding and an OA position.
**R**ecommendation	As risk factors for impacted fetal head are present, the operating obstetrician should prepare the rest of the team by providing some information from the SBAR before starting the simulated caesarean birth. For the purpose of this simulation, the abdominal and uterine incisions have already been made.

van der Scheer JW, *et al. BMJ Open Qual* 2024; 12:e002340. doi: 10.1136/bmjoq-2023-002340

‘Pregnant’ actor instructions for the simulation

Prior to the simulation

Position yourself behind the PROMPT Flex® maternal mannequin (which will be either on a theatre table or on a full-size patient couch if the theatre table is not available).

You are Meena. You are 33 years old and have been in labour for a long time. You have just been transferred to theatre as your obstetrician has advised that you have a caesarean birth because your cervix has not dilated further than 9cm over the past four hours. You are quite worried about having a caesarean birth because you had originally planned to give birth in the midwife-led birth centre.

The epidural that was sited during labour remains in your back, and you have been given medication via the epidural catheter to ensure that you remain pain free during your caesarean birth. The cardiotocograph (CTG) that has been used to monitor your baby’s heart rate in labour has recently been discontinued in preparation for your caesarean birth, and you have been reassured that your baby’s heart rate has always been fine.

During the simulation:

After the handover has finished, say “I’m feeling very worried about this caesarean. Is my baby going to be OK?”

Following this, answer questions if they are asked.

Frequently ask what is happening as you can’t see or feel anything.

van der Scheer JW, *et al. BMJ Open Qual* 2024; 12:e002340. doi: 10.1136/bmjoq-2023-002340
