# Supplementary material for: Training for managing impacted fetal head at caesarean birth: multimethod evaluation of a pilot
Source: BMJ Open Qual. 2023 Jul 31;12(3):e002340. doi: 10.1136/bmjoq-2023-002340 (PMC10391817; doi:10.1136/bmjoq-2023-002340)
Supplement: Supplementary data [file bmjoq-2023-002340supp003.pdf]

Supplementary Material 2

Contents

Supplementary Material 2 1

Brief for fieldnotes to record observations that sought to characterise relevant aspects of the observed socio-technical system 2

Post-training focus group discussion guide 3

Questionnaires 5

Pre-training questionnaire (Kirkpatrick Level 2) 5

Post-training questionnaire (Kirkpatrick Level 2) 5

Post-training questionnaire (Kirkpatrick Level 1) 6

Net promotor score of 1 to 10 6

Brief for fieldnotes to record observations that sought to characterise relevant aspects of the observed socio-technical system

Briefing notes

We are interested in **the management of impacted fetal head as part of the whole socio-technical system** so we want to note things like how the team works together, professional roles/boundaries, communication (or lack of it) with other team members and with the woman/partner, the atmosphere during the event etc.

Is there anything that surprises/concerns you during the training/simulation?

Please aim to jot down field notes during the simulation itself and then write/audio record a fuller account as soon as possible afterwards.

We plan to use the field notes as ‘early intelligence’ and then alongside the analysis of the other data (e.g. the transcript of the debriefing recording etc).

van der Scheer JW, *et al. BMJ Open Qual* 2024; 12:e002340. doi: 10.1136/bmjoq-2023-002340

Post-training focus group discussion guide

Questions focus on healthcare professionals, but with opportunity to involve MVP volunteers in all questions and discussion. The second question also has specific prompts for MVPs. The audio of the debrief session will be used for further analysis. Depending on the time available, some or all of the following can be used, with appropriate prompts/follow up questions – the order of the questions is not set.

THIS team member to 'verbally label' the audio recording of the session using the following script: "This is a debriefing for the ABC Phase 2 programme, pilot testing and evaluation of the impacted fetal head training. I am XX facilitating the debriefing today at [insert hosp name] hospital. The time is XXX on [date].

For the sake of the recording, ask everyone to introduce themselves by role (not by name) and with a participant number, e.g.:

Facilitators (interviewers)

- "I'm the ABC team midwife facilitating the sim and I'm facilitator number 1"
- "I'm the THIS institute team member facilitating the debriefing and I'm facilitator number 2"

Participants

- "I'm a midwife and I'm participant number 1"
- "I'm a midwife coordinator and I'm participant number 2"
- "I'm acting the woman in labour and I'm participant number 3" etc.

Thank everyone for their involvement and acknowledge that it's a somewhat strange setting to be in. Reiterate that the purpose of the simulations today is not to evaluate anyone's clinical judgement or skills, but to evaluate how we can optimise the current training package. Explain that we'll be audio recording this session. As with the video recordings we will be storing the recordings securely and only using them for the purposes of exploring how to improve the prototype tool. Any quotations will be anonymised.

- **What for you were the best/most helpful parts of the training session? And the worst/least helpful?**
  <u>*Optional prompts:*</u>
    - *Was there anything missing that you think should be added to the training? And anything you would take out?*
    - *If money and time were no object, what should this training look like?*
    - *Are there any 'take home' resources that you would like following this training?*
    - *Is there anything that you would recommend that your colleagues do before coming on the training? What would that be? (e.g. prior reading, check unit policy etc)*
    - *How useful did you find this training for your practice around managing impacted fetal head?*
    - *Was there anything that surprised you about the session?*
    - *Is there one key message that you would like the ABC team to take away in improving the training?*

- **Do you think this training on managing impacted fetal head would change how you communicate with the person in labour and their birth partner? How/in what ways?**

  Questions to prompt MVP volunteers more specifically in this discussion:

  - What did the healthcare staff do well when communicating with you during the obstetric emergency?
  - What did the healthcare staff do less well when communicating with you during the obstetric emergency?
  - How would you improve the way the healthcare staff communicated with you during the obstetric emergency?
  - Could you comment on the language used by health professionals during the obstetric emergency?
  - Could you comment on the inclusion of the birth partner [if present]

- **Are there any ways you could see the work of the multidisciplinary team changing as a result of this training?**
  Optional prompts:
  - *For example, would it change communication between team members? In what ways?*

- **What do you think your colleagues would think about this training? What concerns might they have?**
  Optional prompts:
  - *Is that different depending on which professional group is involved – midwives, obstetricians, anaesthetists for example?*
  - *Are there any local factors/circumstances that might help/hinder introducing this training?*

- **If an obstetric emergency with an impacted fetal head happened tomorrow during your shift, how confident would you feel about managing it?** [used in the guide for first two sites only]
  Optional prompts:
  - *If not completely confident, what would make a difference?*

- **Are there any specific comments you would like to make about the training resources used today?**
  Optional prompts:
  - *What did you like about them? What do you think works well?*

Questionnaires

Pre-training questionnaire (Kirkpatrick Level 2)

Statements on a 7-point Likert scale ranging from strongly agree to strongly disagree (strongly agree-agree-somewhat agree-neither agree nor disagree-somewhat disagree-disagree-strongly disagree)

We would like to ask you a few questions about the management of impacted fetal head at caesarean section.

All maternity staff:

- I feel confident about how to manage a situation in which an impacted fetal head at caesarean section is anticipated
- I feel confident that everyone in the clinical team knows when an impacted fetal head at caesarean section has been identified.
- I feel confident that everyone in the clinical team will know exactly what to do in the event of an impacted fetal head at caesarean section.
- I feel confident about my own role in the management of impacted fetal head at caesarean section.
- I feel confident that communication between members of the clinical team during the obstetric emergency of an impacted fetal head will be optimal.
- I feel confident about communicating with the person in labour and their birth partner about impacted fetal head at caesarean section.

Midwives and obstetricians only:

- I feel confident about performing vaginal disimpaction (push-up) (Obstetricians and midwives only)

Obstetricians only:

- I feel confident about diagnosing an impacted fetal head at caesarean section (Obstetricians only)
- I feel confident about performing reverse breech extraction (Obstetricians only)

Post-training questionnaire (Kirkpatrick Level 2)

These questions are the same ones as posed during the pre-training survey.

Statements on a 7-point Likert scale ranging from strongly agree to strongly disagree (strongly agree-agree-somewhat agree-neither agree nor disagree-somewhat disagree-disagree-strongly disagree)

As in the survey before the training, we would like to ask you a few questions about impacted fetal head at caesarean section.

All maternity staff:

- I feel confident about how to manage a situation in which an impacted fetal head at caesarean section is anticipated.
- I feel confident that everyone in the clinical team knows when an impacted fetal head at caesarean section has been identified.
- I feel confident that everyone in the clinical team will know exactly what to do in the event of an impacted fetal head at caesarean section.
- I feel confident about my own role in the management of impacted fetal head at caesarean section.

van der Scheer JW, *et al. BMJ Open Qual* 2024; 12:e002340. doi: 10.1136/bmjoq-2023-002340

- I feel confident that communication between members of the clinical team during the obstetric emergency of an impacted fetal head will be optimal.
- I feel confident about communicating with the person in labour and their birth partner about impacted fetal head at caesarean section.

Midwives and obstetricians only:

- I feel confident about performing vaginal disimpaction (push-up) (Obstetricians and midwives only)

Obstetricians only:

- I feel confident about diagnosing an impacted fetal head at caesarean section (Obstetricians only)
- I feel confident about performing reverse breech extraction (Obstetricians only)

Post-training questionnaire (Kirkpatrick Level 1)

Statements on a 7-point Likert scale ranging from strongly agree to strongly disagree (strongly agree-agree-somewhat agree-neither agree nor disagree-somewhat disagree-disagree-strongly disagree)

We would love to hear a few last things about what you thought about the training. We have ten questions we would like to ask you.

1. This training was **relevant** to my clinical practice in relation to managing impacted fetal head at caesarean section.
2. The training will **help maternity staff better manage** impacted fetal head at caesarean section
3. The training will help to **improve outcomes** following impacted fetal head at caesarean section.
4. The **simulations** helped my learning in the management of impacted fetal head at caesarean section.
5. The **use of augmented reality** helped my learning in managing impacted fetal head at caesarean section.
6. The **animated video** helped my learning in managing impacted fetal head at caesarean section.
7. The **management algorithms** will support my clinical practice or participation in managing impacted fetal head at caesarean section.
8. This **training overall** will improve my clinical practice or participation in managing impacted fetal head at caesarean section.
9. Now that I've had the training, I realise that I had some gaps in my knowledge about managing impacted fetal head at caesarean section.

Net promotor score of 1 to 10

How likely are you to recommend this training to a colleague, on a scale of 1 to 10? A score of 1 means not at all likely to recommend the training, and a score of 10 means extremely likely to recommend it. [participant can select 1,2,3,4,5,6,7,8,9 or 10]
