# Supplementary material for: Training for managing impacted fetal head at caesarean birth: multimethod evaluation of a pilot
Source: BMJ Open Qual. 2023 Jul 31;12(3):e002340. doi: 10.1136/bmjoq-2023-002340 (PMC10391817; doi:10.1136/bmjoq-2023-002340)
Supplement: Supplementary data [file bmjoq-2023-002340supp001.pdf]

Supplementary tables S1 and S2

Table S1. Number and percentage of participants responding to post-training questionnaire statements on relevance and helpfulness of the training for anticipating, identifying and management impacted fetal head (IFH).

	Strongly agree	Agree	Somewhat agree	Neither agree nor disagree	Somewhat disagree	Disagree	Strongly disagree
This training was relevant to my clinical practice in relation to managing IFH at caesarean section	45 (79%)	11 (19%)	1 (2%)	0 (0%)	0 (0%)	0 (0%)	0 (0%)
The training will help to improve outcomes following IFH at caesarean section	41 (72%)	14 (25%)	1 (2%)	1 (2%)	0 (0%)	0 (0%)	0 (0%)
The training will help maternity staff better manage IFH at caesarean section	48 (84%)	7 (12%)	1 (2%)	0 (0%)	0 (0%)	1 (2%)	0 (2%)
The simulations helped my learning in the management of IFH at caesarean section	49 (86%)	8 (14%)	0 (0%)	0 (0%)	0 (0%)	0 (0%)	0 (0%)
The use of augmented reality helped my learning in managing IFH at caesarean section	27 (47%)	13 (23%)	11 (19%)	4 (7%)	2 (4%)	0 (0%)	0 (0%)
The animated video helped my learning in managing IFH at caesarean section	42 (74%)	13 (23%)	2 (4%)	0 (0%)	0 (0%)	0 (0%)	0 (0%)
The management algorithms will support my clinical practice or participation in managing IFH at caesarean section	29 (51%)	26 (46%)	2 (4%)	0 (0%)	0 (0%)	0 (0%)	0 (0%)
This training overall will improve my clinical practice or participation in managing IFH at caesarean section	44 (77%)	12 (21%)	0 (0%)	1 (2%)	0 (0%)	0 (0%)	0 (0%)

Table S2. Number and percentage of participants responding to pre-training and post-training questionnaire statements on confidence in knowledge and relating to anticipating, identifying and management impacted fetal head (IFH).

	Strongly agree	Agree	Somewhat agree	Neither agree nor disagree	Somewhat disagree	Disagree	Strongly disagree	Mean*	Post-pre mean*
	I feel confident about performing vaginal disimpaction ("push-up") (obstetricians and midwives only, n=46)								
Pre	2 (4%)	18 (39%)	7 (15%)	8 (17%)	4 (9%)	7 (15%)	0 (0%)	4.67	
Post	22 (48%)	22 (48%)	2 (4%)	0 (0%)	0 (0%)	0 (0%)	0 (0%)	6.43	+1.76
	I feel confident about diagnosing an IFH at caesarean section (obstetricians only, n=25)								
Pre	10 (40%)	13 (52%)	2 (8%)	0 (0%)	0 (0%)	0 (0%)	0 (0%)	6.32	
Post	16 (64%)	9 (36%)	0 (0%)	0 (0%)	0 (0%)	0 (0%)	0 (0%)	6.64	+0.32
	I feel confident about performing reverse breech extraction (obstetricians only, n=25)								
Pre	4 (16%)	11 (44%)	4 (16%)	3 (12%)	2 (8%)	1 (4%)	0 (0%)	5.32	
Post	9 (36%)	14 (56%)	2 (8%)	0 (0%)	0 (0%)	0 (0%)	0 (0%)	6.28	+0.96
	I feel confident about how to manage a situation in which an IFH at caesarean section is anticipated (all participants, n=57)								
Pre	4 (7%)	22 (39%)	13 (23%)	8 (14%)	2 (4%)	6 (11%)	2 (4%)	4.86	
Post	30 (53%)	25 (44%)	2 (4%)	0 (0%)	0 (0%)	0 (0%)	0 (0%)	6.49	+1.63
	I feel confident that everyone in the clinical team knows when an IFH at caesarean section has been identified (all participants, n=57)								
Pre	3 (5%)	7 (12%)	14 (25%)	10 (18%)	14 (25%)	8 (14%)	1 (2%)	4.07	
Post	20 (35%)	24 (42%)	11 (19%)	2 (4%)	0 (0%)	0 (0%)	0 (0%)	6.08	+2.01

van der Scheer JW, *et al. BMJ Open Qual* 2024; 12:e002340. doi: 10.1136/bmjoq-2023-002340

	I feel confident that everyone in the clinical team will know exactly what to do in the event of an IFH at caesarean section (all participants, n=57)								
Pre	0 (0%)	3 (5%)	15 (26%)	7 (12%)	19 (33%)	11 (19%)	2 (4%)	3.54	
Post	18 (32%)	28 (49%)	8 (14%)	2 (4%)	1 (2%)	0 (0%)	0 (0%)	6.05	+2.51
	I feel confident about my own role in the management of IFH at caesarean section (all participants, n=57)								
Pre	9 (16%)	21 (37%)	10 (18%)	10 (18%)	4 (7%)	1 (2%)	2 (4%)	5.17	
Post	42 (74%)	14 (25%)	0 (0%)	1 (2%)	0 (0%)	0 (0%)	0 (0%)	6.70	+1.53
	I feel confident that communication between members of the clinical team during the obstetric emergency of an IFH will be optimal (all participants, n=57)								
Pre	3 (5%)	8 (14%)	23 (40%)	8 (14%)	10 (18%)	4 (7%)	1 (2%)	4.47	
Post	24 (42%)	25 (44%)	6 (11%)	2 (4%)	0 (0%)	0 (0%)	0 (0%)	6.24	+1.77
	I feel confident about communicating with the person in labour and their birth partner about IFH at caesarean section (all participants, n=57)								
Pre	3 (5%)	20 (35%)	16 (28%)	5 (9%)	9 (16%)	3 (5%)	1 (2%)	4.82	
Post	28 (49%)	26 (46%)	3 (5%)	0 (0%)	0 (0%)	0 (0%)	0 (0%)	6.43	+1.61

*Mean calculated based on the following replacements: strongly agree = 7, agree = 6, somewhat agree = 5, neither agree nor disagree = 4, somewhat disagree = 3, disagree = 2, strongly disagree = 1.
